# Synthesis and COX-2 Inhibitory Activity of 4-[(*E*)-2-(4-Oxo-3-phenyl-3,4-dihydroquinazolin-2-yl)ethenyl]benzene-1-sulfonamideand Its Analogs

**DOI:** 10.3390/ph5121282

**Published:** 2012-11-27

**Authors:** Hayun, Sumi Hudiyono, Muhammad Hanafi, Arry Yanuar

**Affiliations:** 1 Faculty of Pharmacy, University of Indonesia, Depok 16424, West Java, Indonesia; Email: hayun.ms@ui.ac.id (H.); arry.yanuar@ui.ac.id (A.Y.); 2 Department of Chemistry, Faculty of Natural Sciences, University of Indonesia, Depok 16424, West Java, Indonesia; Email: hudiyono@ui.ac.id (S.H.); 3 Research Center for Chemistry, Indonesian Institute of Sciences, Serpong 15314, West Java, Indonesia; Email: hanafi124@yahoo.com (M.H.)

**Keywords:** quinazolin-4-one, benzenesulfonamide, COX-2 inhibitor

## Abstract

Some novel 3-phenyl-2-[(*E*)-2-phenylethenyl]-3,4-dihydroquinazolin-4-one derivatives possessing *para*-sulfonamides groups on the phenyl ring of the 2-phenylethenyl moiety have been synthesized and their COX-2 inhibitory activity evaluated. The stuctures of the synthesized compounds were confirmed on the basis of FT-IR, ^1^H-NMR, ^13^C-NMR and mass spectral data. The COX-2 inhibition screening assay revealed that 4-[(*E*)-2-{3-(4-methoxyphenyl)-4-oxo-3,4-dihydroquinazolin-2-yl}ethenyl]benzene-1-sulfonamide had a maximum COX-2 inhibition (47.1%), at a concentration of 20 μM.

## 1. Introduction

**Figure 1:**
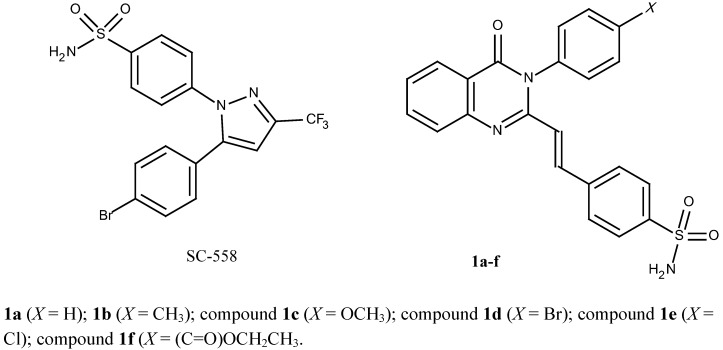
Chemical structure of SC-558 [5] and general formula of the title compounds **1a-f**.

Compounds containing the 4(3*H*)-quinazolinone ring system possess various biological activities [1]. Some 2,3-diaryl-4(3H)-quinazolinone derivatives exhibit COX-2 inhibitory and anti-inflammatory activity [2,3,4]. The majority of COX-2 inhibitors are diaryl heterocycles. The presence of *para*-sulfonamides or *para*-sulfonylmethanes on one of the aryl rings was found to be essential for optimum COX-2 selectivity and inhibitory potency, while a wide variety of heterocycles, in general a five membered or six membered ring, can be used as the central ring system [5,6]. The results of a molecular docking study showed that 2,3-diaryl-4(3H)-quinazolinones possessing *p*-benzene-sulfonamide moieties at C-2 and phenyl rings at N-3 were predicted to have potent COX-2 inhibitory activity. The study used SC-558 (Figure 1) as reference ligand [7]. As continuation of our research program, we report herein the synthesis and COX-2 inhibitory activity evaluation of 4-[(*E*)-2-(4-oxo-3-phenyl-3,4-dihydroquinazolin-2-yl)ethenyl]benzene-1-sulfonamide and its analogs **1a-f** (Fig.1).

## 2. Results and Discussion

**Scheme 1:**
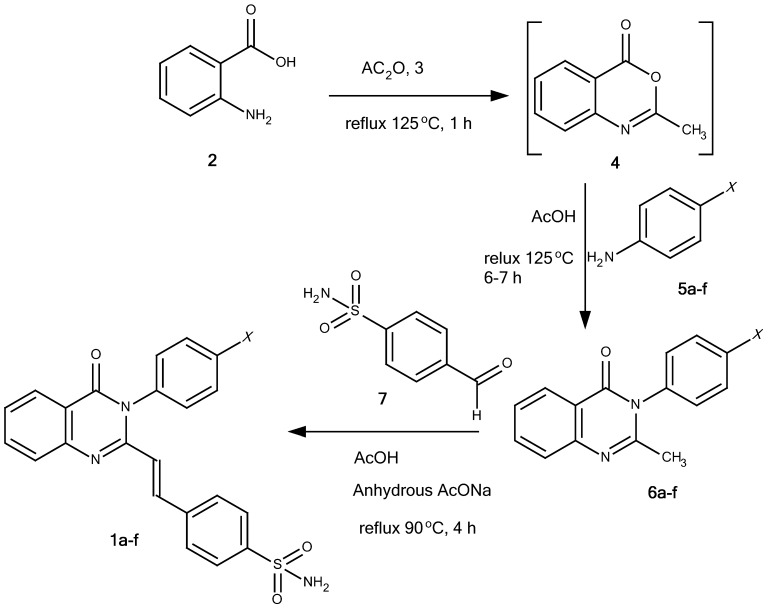
Synthesis of the title compounds **1a-f**.

**Scheme 2:**
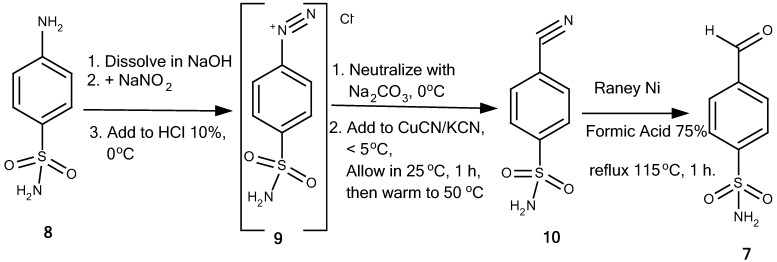
Synthesis of 4-formylbenzenesulfonamide (**7**).

The title compounds **1a-f** were synthesized stepwise by the method summarized in Scheme 1 and Scheme 2. Anthranilic acid (**2**) was reacted with acetic anhydride (**3**) at reflux temperature for 1 hour to provide benzoxazinone **4** [8]. Treatment of **4** with corresponding anilines **5a-f** in glacial acetic acid under reflux conditions for 6-7 hours gave 2-methyl-3-phenyl-4(3*H*)-quinazolinones **6a-f** [1,8,9]. 4-Formylbenzenesulfonamide (**7**) was synthesized from sulfanilamide (**8**). Diazotization of **8** led to diazonium salt **9**, which was then neutralized with sodium carbonate and added dropwise into CuCN/KCN (Sandmeyer reaction) to yield benzonitrile **10**. Reduction of **10** with Raney nickel alloy in 75% aqueous formic acid gave 4-formylbenzenesulfonamide (**7**) [10]. Finally, the condensation of **6a-f** and **7** in the presence of anhydrous sodium acetate as catalyst and glacial acetic acid as solvent at 90 °C for 4 hours (TLC monitoring) afforded the title compounds **1a-f** [11,12].

The IR spectra of compounds **1a-f** showed absorption bands at 3,331–3,365 and 3,200–3,265 cm^-1^ due to the presence of the NH_2_ group. The bands at 1,330–1,340 and 1,163–1,166 cm^-1^ correspond to ‑SO_2_-, while the carbonyl groups of the quinazolinones are observed as strong bands at 1,654–1,691 cm^-1^. In the ^1^H-NMR spectra the two protons of the sulfonamide NH_2_ group appear as a broad singlet at δ 7.40 ppm, while the presence of the two protons of the –(*E*)-ethenyl chain of the compounds are observed as doublets at 6.43–6.51 ppm (1H, *J* = 15,0 Hz) and 7.87–7.91 (1H, *J* = 15,0 Hz), respectively. The structures were further supported by ^13^C-NMR and HR-ESI-MS of the compounds which showed the complete agreement with the assigned molecular structures. 

The results of the COX-2 inhibition screening assay of the compounds (at concentrations of 10, 20 and 50 μM) are listed in Table 1. Compound **1a** is inactive as a COX-2 inhibitor, while compounds **1b-f** are active. These data indicated that the substituent at the *para*-position of the 3-phenyl rings is essential for their inhibitory activity. In the present series, 4-[(*E*)-2-{3-(4-methoxyphenyl)-4-oxo-3-4-dihydroquinazolin-2-yl}ethenyl]benzene-1-sulfonamide (**1c**) showed a maximum COX-2 inhibition (47.1%) at a concentration of 20 μM. All our compounds are less potent COX-2 inhibitors than celecoxib (which showed 80.1% inhibition at a concentration of 1 μM), but the COX-2 inhibitory activity of the most active compound is somewhat higher than that of 2-(4-nitrophenyl)-3-(tolyl)-4(3*H*)-quinazolinone reported earlier (which showed a maximum COX-2 inhibition of 27.72% at a concentration of 22 μM) [4]. The solubility of the compounds in the assay system used is poor, and at concentrations of 50 μM the compounds do not dissolve completely. 

**Table 1:** *In vitro* COX-2 inhibition data (%) for compounds **1a-f** (see Figure 1).

Compd	*X*	Inhibition ± SD (%)^1)^ at concentration of		
		10 μM	20 μM	50 μM
**1a**	H	ns	ns	ns
**1b**	CH_3_	21.2 ±1.4	17.4 ±1.3	30.6 ±2.7
**1c**	OCH_3_	39.1 ±0.9	47.1 ±4.3	38.2 ±2.3
**1d**	Br	29.2 ±1.2	36.3 ±3.6	33.7 ±2.3
**1e**	Cl	ns	27.2 ±1.8	38.4 ±3.8
**1f**	COOC_2_H_5_	10.8 ±0.5	36.7 ±1.6	33.9 ±3.0
**Reference compound**		**Inhibition ± SD (%)^1)^ at ** **1 μM concentration**		
Celecoxib		80.1 ±2.7		

Values are the mean (*n* = 3) and SD (Standard deviation) of the % inhibition acquired using COX inhibition assay kit (Catalog No. 560131, Cayman Chemical Co., Ann Arbor, MI, USA); ns = not significant (%-inhibition <10%).

## 3. Experimental

### 3.1. Chemistry. General Procedures

All solvents, chemicals, and reagents were obtained commercially and used without purification. Purity tests of the products was performed by the TLC method on silica gel 60 F254 plates (Merck). Melting points were determined in the capillary tube using melting point apparatus (Stuart Scientific) and are uncorrected. Infrared (IR) spectra were recorded on a FTIR spectrophotometer (8400S, Shimadzu), ^1^H-NMR and ^13^C-NMR spectra were recorded on a JEOL JNM 500 spectrometer, using TMS as internal standard, and high resolution mass spectra (HRMS) were measured with a Waters LCT Premier XE (ESI-TOF) system in positive mode. 

*4-[(E)-2-(4-oxo-3-phenyl-3,4-quinazolin-2-yl)ethenyl]benzene-1-sulfonamide* (**1a**): A mixture of 2-methyl-3-phenyl-4(3*H*)-quinazolinone (**6a**; 0.98 g, 5 mmol), 4-formylbenzenesulfonamide (**7**; 1.02 g, 5.5 mmol) and anhydrous sodium acetate (1 g) were dissolved in glacial acetic acid (6 mL) and refluxed at 100 °C. The progress of the reaction was monitored by TLC. After the reaction was completed, the reaction mixture was poured onto cold water and filtered off through a Buchner funnel. The solid product was washed with cold water, recrystallized from suitable solvents and dried in vacuum oven at 85 °C for 1 hour to provide **1a** as pale yellow crystalline powder (57.9% yield, recrystallized from acetonitril-water, and washed with cold ethanol), m.p. 277-278 °C. IR (KBr), □_max_, cm^-1^: 3,362, 3,234 (primary sulfonamide N-H streching), 3,049 (aromatic/alkene C-H streching), 1,672 (C=O lactam), 1,556 (C=C), 1,340, 1,165 (sulfonamide asymmetric and symmetric SO_2_ streching). ^1^H-NMR (DMSO-d_6_), δ/ppm: 8.16 (1H, dd, *J* = 7.1, 1.9 Hz, 5-H_quinazolinone_), 7.88 (1H, td, *J* = 7.1, 1.9 Hz, 7-H_quinazolinone_), 7.87 (1H, d, *J* = 15.5 Hz, 2-H_trans ethenyl_), 7.80 (1H, d, *J* = 7.8 Hz, 8-H_quinazolinone_) 7.74 (2H, d, *J* = 8.4 Hz, 2”,6”-H_Ar_), 7.53-7.63 (6H, m, overlap of 6-H_quinazolinone_, 3”,5”; 2’,6’; and 4’-H_Ar_), 7.48 (2H, d, *J* = 8.45 Hz, 3’,5’-H_Ar_), 7.39 (2H, s broad, NH_2 sulfonamide_), and 6.43 (1H, d, *J* = 15.5 Hz, 1-H_trans ethenyl_). ^13^C-NMR (DMSO-d6, 125 MHz, TMS), δ/ppm: 161.2 (C(=O)-N), C-4_quinazolinone_, 151.1 (N-C=N-, C-2_quinazolinone_ ), 147.3 (C_Phe-N=C_, C-9_quinazolinone_), 144.5 (C-1”_Phe-1”-sulfonamide_), 138.0, 137.1, 136.8, 134.9, 130.0, 129.7, 129.3, 127.9, 127.3, 126.9, 126.5, 126.3, 122.6, and 120.8 (C aromatic and C ethenyl)**. **HRESIMS (*m/z*): found 404.1046 ([M+H]^+^), calculated masses of C_22_H_18_N_3_O_3_S: 404.1069 (error 5.7 ppm). 

*4-[(E)-2-{3-(4-methylphenyl)-4-oxo-3,4-dihydroquinazolin-2-yl}ethenyl]benzene-1-sulfonamide* (**1b**)**:** Compound **1b** was prepared as a pale yellow crystalline powder from 2-methyl-3-(4-methylphenyl)-4(3*H*)-quinazolinone (**6b**) using the procedure described for **1a** (35.8% yield, recrystallized from THF-ethanol, followed by chloroform), m.p. 244-245 °C. IR (KBr), □_max_, cm^-1^: 3,329, 3,265 (primary sulfonamide N-H streching), 3,063 (aromatic/alkene C-H streching), 1,691 (C=O lactam), 1,550 (C=C), 1,340, 1,165 (sulfonamide asymmetric and symmetric SO_2_ streching). ^1^H-NMR (DMSO-d_6_) δ/ppm: 8.14 (1H, dd, *J* = 7.8; 1.3 Hz, 5-H_quinazolinone_), 7.90 (1H, td, *J* = 8.9, 1.9 Hz, 7-H_quinazolinone_), 7.89 (1H, d, *J* = 15.5 Hz, 2-H_trans ethenyl_), 7.80 (1H, d, *J* = 6 Hz, 8-H_quinazolinone_), 7.78 (2H, d, *J* = 8.4 Hz, 2”,6”-H_Phe-1”-sulfonamide_), 7.56 (1H, td, *J* = 6.5; 1.3 Hz, 6-H_quinazolinone_), 7.53 (2H, d, *J* = 8.5 Hz, 3”,5”-H_Phe-1”-sulfonamide_), 7.42 (2H, s, NH_2sulfonamide_), 7.37 (2H, d, *J* = 8.45 Hz, 2’,6’-H_Ar_), 7,34 (2H, d, *J* = 7.8 Hz, 3’,5’-H_Ar_), 6,43 (1H, d, *J* = 15.5 Hz, 1-H_trans ethenyl_), 2,44 (3H, s, CH_3_-Ar). ^13^C-NMR (DMSO-d_6_) δ/ppm: 161.2 ((C(=O)-N), C-4_quinazolinone_), 151.1 (N-C=N-, C-2_quinazolinone_), 147.2 (C_Phe-N=C_, C-9_quinazolinone_), 144.5 (C-1”_Phe-1”-sulfonamide_), 138.7, 138.0, 137.1, 136.8, 134.9, 134.8, 130.1, 128.6, 127.9, 127.3, 126.8, 126.3, 122.6, 120.7 (C aromatic and C ethenyl), and 20.4 (CH_3_-Ar). HRESIMS (*m/z*): found 418.1237 ([M+H]^+^), calculated masses of C_23_H_20_N_3_O_3_S: 418.1225 (error 2.9 ppm).

*4-[(E)-2-{3-(4-methoxyphenyl)-4-oxo-3,4-dihydroquinazolin-2-yl}ethenyl]benzene-1-sulfonamide* (**1c**)**:** Compound **1c** was prepared from 2-methyl-3-(4-methoxyphenyl)-4(3*H*)-quinazolinone (**6c**) using the procedure described for **1a** as a pale yellow crystalline powder (45.5% yield, recrystallized from ethanol), m.p. 228-229 °C. IR (KBr), □_max_, cm^-1^: 3,365, 3,246 (primary sulfonamide N-H streching), 3,080 (aromatic/alkene C-H streching), 1,675 (C=O lactam), 1,555 (C=C), 1,338, 1,163 (sulfonamide asymmetric and symmetric SO_2_ streching) and 1,250 (Ar-O-Al ether). ^1^H-NMR (DMSO-d_6_) δ/ppm: 8.14 (1H, dd, *J* = 7.8; 1.3 Hz, 5-H_quinazolinone_), 7.88 (1H, td, *J* = 7.0; 1.9 Hz, 7-H_quinazolinone_), 7,90 (1H, d, *J* = 15.6 Hz, 2-H_trans ethenyl_), 7.77-7.80 (3H, overlap, d, *J* = 8.4 Hz, H_Ar_), 7.55 (1H, t, *J* = 7.9 Hz, 6-H_quinazolinone_), 7.58 (2H, d, *J* = 8.5 Hz (3”,5”-H_Phe-1”-sulfonamide_), 7,39 (2H, d, *J* = 6.5 Hz, (2’,6’-H_Phe-4’-OMe_), 7.34 (2H, d, *J* = 7.2 Hz, (3’,5’-H_Phe-4’-OMe_)), 7,42 (2H, s, NH_2sulfonamide_), 6,51 (1H, d, *J* = 15.6 Hz, 1-H_trans ethenyl_) and 3.86 (3H, s, CH_3_O-). ^13^C-NMR (DMSO-d_6_) δ/ppm: 161.4 ((C(=O)-N), C-4_quinazolinone_), 159.5 (C-4’_Phe-O_), 151.5 (N-C=N-, C-2_quinazolinone_), 147.3 (C_Phe-N=C_, C-9_quinazolinone_), 144.5 (C-1”_Phe-1”-sulfonamide_), 138.0, 136.9, 134.7, 130.0, 129.2, 127.9, 127.3, 126,8, 126.5, 126,3, 122.7, 120.7, 114.8 (C aromatic and C ethenyl), and 55.4 (methoxy). HRESIMS (*m/z*): found 434.1176 ([M+H]^+^), calculated masses of C_23_H_20_N_3_O_4_S: 434.1175 (error 0.2 ppm).

*4-[(E)-2-{3-(4-bromophenyl)-4-oxo-3,4-dihydroquinazolin-2-yl}ethenyl]benzene-1-sulfonamide* (**1d**): Compound **1d** was prepared from 2-methyl-3-(4-bromophenyl)-4(3*H*)-quinazolinone (**6d**) using the procedure described for **1a** as a pale yellow crystalline powder (41.2% yield, recrystallized from ethanol), m.p. 211-212 °C. IR (KBr), □_max_, cm^-1^: 3,335, 3,236 (primary sulfonamide N-H streching), 3,095 (aromatic/alkene C-H streching), 1,683 (C=O lactam), 1,556 (C=C), 1,338, 1,165 (sulfonamide asymmetric and symmetric SO_2_ streching). ^1^H-NMR (DMSO-d_6_) δ/ppm: 8.14 (1H, dd, *J* = 7.8; 1.3 Hz, 5-H_quinazolinone_), 7.88 (1H, td, *J* = 7.0; 1.3 Hz, 7-H_quinazolinone_), 7.93 (1H, d, *J* = 15.0 Hz, 1H, 2-H_trans ethenyl_), 7.78-7.83 (5H, m, overlap, H_Ar_), 7.63 (2H, d, *J* = 8.5 Hz, 2’,6’-H _Phe-4’-Br_), 7.57 (1H, t, *J* = 7.8 Hz, 6-H_quinazolinone_), 7.48 (2H, d, *J* = 7.2 Hz, 3’,5’-H _Phe-4’-Br_), 7.4 (2H, s, NH_2 sulfonamide_), and 6.5 (1H, d, *J* = 15.0 Hz, 1-H_trans ethenyl_). ^13^C-NMR (DMSO-d_6_) δ/ppm: 161.1 ((C(=O)-N), C-4_quinazolinone_), 150.9 (N-C=N-, C-2_quinazolinone_), 147.2 (C_Phe-N=C_, C-9_quinazolinone_), 144.6 (C-1”_Phe-1”-sulfonamide_), 137.9, 137.4, 136.1, 134.9, 132.7, 131.3, 128.1, 127.3, 126.9, 126.3, 120.6, 122.5, 122.4 (C aromatic and C ethenyl). HRESIMS (*m/z*): found 482.0164 ([M+H]^+^, 95%) and 484.0157 ([M+H]^+^, 100%), calculated masses of C_22_H_17_N_3_O_3_SBr: 482.0174 (error 2.1 ppm).

*4-[(E)-2-{3-(4-chlorophenyl)-4-oxo-3,4-dihydroquinazolin-2-yl}ethenyl]benzene-1-sulfonamide* (**1e**): Compound **1e** was prepared from 2-methyl-3-(4-chlorophenyl)-4(3*H*)-quinazolinone (**6e**) using the the procedure described for **1a** as a pale yellow crystalline powder (49.5% yield, recrystallized from THF-ethanol, followed chloroform), m.p. 248-249 °C. IR (KBr), □_max_, cm^-1^: 3340, 3200 (primary sulfonamide N-H streching), 3066 (aromatic/alkene C-H streching), 1672 (C=O lactam), 1552 (C=C), and 1330, 1166 (sulfonamide asymmetric and symmetric SO_2_ streching). ^1^H-NMR (DMSO-d_6_) δ/ppm: 8.14 (1H, dd, *J* = 7.8; 1.3 Hz, 5-H_quinazolinone_), 7.88 (1H, td, *J* = 7.8; 1.3 Hz, 7-H_quinazolinone_), 7.91 (1H, d, *J* = 15,0 Hz, 2-H_trans ethenyl_), 7.8 (1H, t, *J* = 8 Hz, 6-H_quinazolinone_), 7.79 (2H, d, *J* = 8.4 Hz, 2”,6”-H_Phe-1”-sulfonamide_), 7.68 (2H, d, *J* = 8.5 Hz, 3”,5”H-_Phe-1”-sulfonamide_), 7.63 (2H, d, *J* = 7.8 Hz, 2’,6’H_-Phe-4’-Cl_), 7.54 (2H, d, *J* = 10 Hz, 3’,5’-H_Phe-4’-Cl_), 7.4 (2H, s, NH_2 sulfonamide_), 7.57 (1H, d, *J* = 7.15 Hz, 8-H_quinazolinone_), 6.5 (1H, d, *J* = 15.0 Hz, 1-H_trans ethenyl_). ^13^C-NMR (DMSO-d_6_) δ/ppm: 161.2 ((C(=O)-N), C-4_quinazolinone_), 150.9 (N-C=N-, C-2_quinazolinone_), 147.2 (C_Phe-N=C_, C-9_quinazolinone_), 144.6 (C-1”_Phe-1”-sulfonamide_), 137.9, 137.4, 135.7, 134.9, 133.3, 130.9, 129.7, 128.1, 127.3, 126.9, 126.5, 126.3, 120.7 (C aromatic and C ethenyl). HRESIMS (*m/z*): found 438.0674 ([M+H]^+^, 100%), 440,0647 ([M+H]^+^, 40%), calculated masses of C_22_H_17_N_3_O_3_SCl: 438,0679 (error 1.1 ppm).

*Ethyl 4-{4-okso-2-[(E)-2-(4-sulfamoylphenyl)ethenyl]-3,4-dihydroquinazolin-3-yl}benzoate* (**1f**)**: **Compound **1f** was prepared from ethyl 4-(2-methyl-4-oxo-3,4-dihydroquinazolin-3-yl)benzoate (**6f**) using the procedure described for **1a** as a pale yellow crystalline powder (50,2% yield, recrystallized from THF-ethanol, and washed with cold ethanol), m.p. 239-240 °C. IR (KBr), □_max_, cm^-1^: 3,331, 3,219 (primary sulfonamide N-H streching), 3,100 (aromatic/alkene C-H streching), 2,960-1,983 (alipatic C-H streching), 1,701 (C=O ester), 1,654 (C=O lactam), 1,556 (C=C), and 1,340, 1,165 (sulfonamide asymmetric and symmetric SO_2_ streching). ^1^H-NMR (DMSO-d_6_) δ/ppm: 8.15 (1H, dd, *J* = 8.4; 1.3 Hz, 5-H_quinazolinone_), 8.17 (2H, d, *J* = 8.5 Hz, 2’,6’_-Phe-1’-COOEt_), 7.93 (1H, d, *J* = 15.5 Hz, 2-H_trans ethenyl_), 7.91 (1H, td, *J* = 7.1; 1.9 Hz, 7-H_quinazolinone_), 7.81 (1H, d, *J* = 7.8 Hz, 8-H_quinazolinone_), 7.76 (2H, d, *J* = 8.5 Hz, 3”,5”-H-_Phe-4”-sulfonamide_), 7.66 (2H, d, *J* = 6.5 Hz, 3’,5’_-_H_Phe-1’-COOEt_), 7.61 (2H, d, *J* = 7.8 Hz, H-2”,6”-_Phe-4”-sulfonamide_ ), 7.57 (1H, t, *J* = 8.5 Hz, 6-H_quinazolinone_), 7.39 (2H, s, NH_2 sulfonamide_), 6.45 (1H, d, *J* = 15.5 Hz, 1-H_trans ethenyl_). ^13^C-NMR (DMSO-d_6_) δ/ppm: 165.1 (C=O ester), 161.1 (C(=O)-N), C-4_quinazolinone_), 150.6 (N-C=N-, C-2_quinazolinone_), 147.2 (C_Phe-N=C_, C-9_quinazolinone_), 144.6 (C-1’-_Phe-1’-COOEt_), 141.0 (C-4”_Phe-4”-sulfonamide_), 137.6, 137.5, 134.9, 130.5, 130.4, 129.6, 128.1, 127.3, 127.0, 126.5, 126.2, 120.6 (C aromatic and C ethenyl), 61.1 (CH_2_-O-) and 14.1 (CH_3_-C). HRESIMS (*m/z*): found 476.1278 ([M+H]^+^), calculated masses of C_25_H_22_N_3_O_5_S: 476.1280 (error 0.4 ppm).

### 3.2. In Vitro Cyclooxygenase-2 (COX-2) Inhibition Assays

The ability of the test compounds **1a-f** listed in Table 1 to inhibit the ability of COX-2 to catalyze the conversion of arachidonic acid to prostaglandin H_2_ (PGH_2_) was determined using a COX inhibitor screening assay kit (catalog No. 560131, Cayman Chemical Co., Ann Arbor, MI, USA) according to the manufacturer’s instructions. Celecoxib was used as reference compound. The test compounds were dissolved in DMSO and added 20 μL to COX reaction tube to get final concentration mentioned at Table 1. 

## 4. Conclusions

A series of 3-phenyl-2-[(*E*)-2-phenylethenyl]-3,4-dihydroquinazolin-4-ones possessing *para*-sulfonamide groups on the phenyl rings of the 2-phenylethenyl moiety and various substituents (*X*= H, CH_3_, OCH_3_, Br, Cl, COOC_2_H_5_) at the *para-*position of the 3-phenyl rings were synthesized and their COX-2 inhibitory activity evaluated. The compounds having substituents at the *para*-position of the 3-phenyl ring showed COX-2 inhibitory activity. All our compounds are less potent COX-2 inhibitors than celecoxib. 4-[(*E*)-2-{3-(4-Methoxyphenyl)-4-oxo-3,4-dihydroquinazolin-2-yl}ethenyl]benzene-1-sulfonamide showed a maximum COX-2 inhibition (47.1%) at concentration of 20 μM. 
